# Effects of pre-gestational exposure to the stressors and perinatal mirtazapine administration on the excitability of hippocampal glutamate and brainstem monoaminergic neurons, hippocampal neuroplasticity, and anxiety-like behavior in rats

**DOI:** 10.1038/s41380-025-03161-3

**Published:** 2025-08-25

**Authors:** Ruslan Paliokha, Mireia Viñas-Noguera, Stanislava Bukatová, Daniil Grinchii, Jana Gaburjáková, Marta Gaburjáková, Hande Özbaşak, Roman Dekhtiarenko, Talah Khoury, Ľubica Lacinová, Eliyahu Dremencov, Michal Dubovický

**Affiliations:** 1 Institute of Molecular Physiology and Genetics, Center of Biosciences, Slovak Academy of Sciences, Bratislava, Slovakia; 2 Institute of Experimental Pharmacology and Toxicology, Center of Experimental Medicine, Slovak Academy of Sciences, Bratislava, Slovakia

**Keywords:** Neuroscience, Depression

## Abstract

When accompanied by excessive exposure to the stressors, pregnancy may result in prenatal depression, that has in turn negative influence on the offspring’s brain. Mirtazapine, among other antidepressants, is commonly used to treat prenatal depression. Even though mirtazapine is generally considered safe for pregnant women, its effect on the offspring brain have not been sufficiently investigated. The present study aimed to examine the effects of chronic unpredictable stress (CUS) in pregestational rats, perinatal mirtazapine treatment, and their combination, on offspring behavior and brain function. We assessed offspring anxiety levels during the elevated plus maze (EPM) test, the expression of pro-neuroplastic proteins in the offspring brain, the excitability of brainstem monoamine and hippocampal glutamate neurons, and the expression and activity of ryanodine receptors (RyR2). Surprisingly, maternal pregestational stress induced an anxiolytic-like effect in the offspring. This anxiolytic effect was associated with an increased excitability of serotonin (5-HT) neurons and detected in the offspring of the vehicle-, but not mirtazapine-treated dams. Perinatal mirtazapine, however, elevated expression of the brain-derived neurotrophic factor (BDNF); this effect was detected in the female offspring of the stressed dams. Regarding the offspring glutamate and dopamine neurons, the combination of maternal stress and mirtazapine inhibited their burst firing, potentially, due to decreased expression of the glutamate receptors. Even though calcium signaling is important for the burst firing of the neurons, the effects of maternal stress and mirtazapine on the burst activity of the offspring glutamate and dopamine might not be mediated via mechanism(s) involving the RyR2. Summarizing, mirtazapine may diminish the negative influence of maternal stress and depression on the offspring brain, via mechanism(s) putatively involving 5-HT neurotransmission and BDNF.

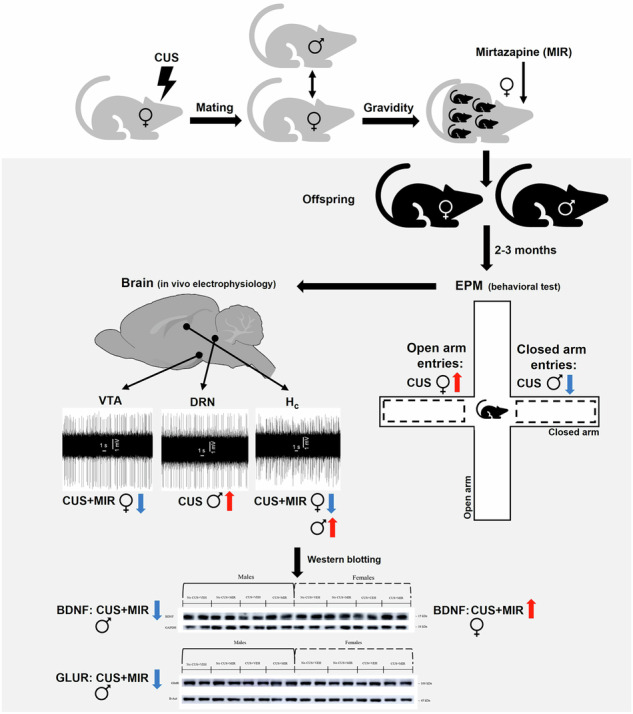

## Introduction

Pregnancy is linked with robust endocrine and neurochemical changes in female organism, rarely observed in other non-pathological conditions. The concentrations of the steroid hormones estradiol and cortisol are gradually increasing during pregnancy, reaching the maximum toward its end. The levels of the brain derived neurotrophic factor (BDNF) are, vice versa, progressively decreasing during pregnancy. BDNF is known to play a key role in the pathophysiology of depression and related mood and anxiety disorders. BDNF is a key regulator of neuroplasticity and neuronal survival as well, and its role in depression is complex and bidirectional. On one hand, reduced BDNF levels are implicated as a contributing factor to depression, as lower neurotrophic support leads to impaired synaptic function and neuronal atrophy [1]. On the other hand, stress and depression themselves downregulate BDNF expression, particularly in the hippocampus and prefrontal cortex, which exacerbates depressive symptoms and hinders recovery [2]. Moreover, genetic variations in the BDNF gene have been linked to susceptibility to depression, further suggesting a causal role of BDNF dysregulation [3]. Importantly, antidepressant treatments have been shown to restore BDNF levels, which correlates with therapeutic efficacy [4]. Likely, due to the decreased BDNF, depressed mood and increased anxiety, not necessarily reaching the level of clinical depression, are frequently experienced during the pregnancy and postpartum [5].

Similarly to pregnancy, stress is generally associated with increased corticosteroids and decreased BDNF. It is thus possible that stress experienced during or before pregnancy may, in certain conditions, lead to neuroendocrine changes extreme enough to induce clinical depression. Indeed, prenatal, and postpartum depression are common complications of pregnancy [6], and excessive exposure to stressors [7, 8] and/or strong decline in BDNF [5] are known risk factors for these disorders.

Prenatal and postpartum depression are common complications of pregnancy, with an estimated prevalence of 15% in high-income countries and up to 25% in low- and middle-income countries [9]. Prenatal depression has been associated with long-term consequences, including altered neurodevelopment, increased risk of mental disorders in offspring, and inflammatory dysregulation that persists into adulthood [10]. Moreover, studies indicate that prenatal depression disrupts brain plasticity, leading to structural changes in key regions such as the amygdala and prefrontal cortex, which may explain the increased susceptibility to anxiety and mood disorders observed in affected offspring [11]. Given these findings, prenatal depression represents a critical period of vulnerability for both mother and child and should be given as much attention as postnatal depression in research and clinical practice.

Depression is known to induce long-lasting changes in the brain, such as decreased neuroplasticity. When depression occurs during pregnancy, it affects not only the maternal, but also embryonal CNS. Increased rates of the attention deficit hyperactivity disorder (ADHD), autism spectrum disorder (AUD), depression, and schizophrenia were indeed observed in children of depressed mothers [12, 13].

Considering the role of stress in the etiology of depression in general and prenatal depression particularly, pregestational exposure of the dams to the chronic unpredictable stress (CUS) has been used as an animal model for prenatal depression. Our previous study [14] showed that pregestational CUS induced long-lasting anhedonia, a behavioral characteristic resembling one of the key symptoms of depression [15]. Dams’ behavioral performance during the elevated plus maze (EPM) test was affected by the pregestational CUS as well [14]. Another study from our team found increased grimace test scores in the dams experienced CUS, suggesting increased nociception; mirtazapine, however, did not have any effect on this characteristic [16].

Pharmacotherapy is a primary treatment strategy in prenatal depression. Literally, all antidepressants act on monoamines, and monoamines play a role in neurogenesis. Since antidepressants pass through the placenta, the putative effects of these drugs on the offspring brain development must be considered. In our previous studies [16–19], we examined the effects of pre-gestational CUS and perinatal treatment with catecholamine releaser bupropion and with α_2_-adrenergic and 5-HT_2A/2C/3_ serotonergic antagonist mirtazapine on the offspring behavioral and neurophysiological characteristics. We found that the maternal CUS resulted in hyperactivity-like behavior and decreased spatial memory in female adolescent offspring [16, 17]. Forced swim test uncovered decreased immobility time in adolescent females and increased swimming in adolescents of both sexes [16]. Elevated plus maze (EPM) test detected increased time spent in closed arms in adolescent males, decreased intersection time in adult males, and decreased number of entries to the open arms in adults of both sexes [16, 19]. Perinatal mirtazapine potentiated hyperactivity- and antidepressant-like effects of the maternal CUS [16]. With respect to the anxiogenic effect of the maternal CUS, both perinatal mirtazapine and bupropion diminished it [19].

Proteins like BDNF, postsynaptic density protein 95 (PSD95), glial fibrillary acidic protein, (GFAP), and glutamate (GLUR) and ryanodine (RyR2) receptors were used in neurobehavioral studies where offspring rats were exposed to CUS and/or treatment with mirtazapine. They are key markers of brain functioning and plasticity. BDNF is crucial for neuronal survival, growth, and synaptic plasticity, often reduced by stress but enhanced by antidepressants. PSD95 is essential for synaptic stability and signaling, reflecting synaptic integrity. GFAP is a marker of astrocyte activation, indicating neuroinflammatory responses to stress. GLUR are critical for excitatory neurotransmission, which is often disrupted by stress. The ryanodine receptor (RyR) is a key intracellular calcium (Ca^2+^) channel, and growing evidence indicates that Ca^2+^ signaling in the brain plays a crucial role in synaptic plasticity [20, 21]. Moreover, Ca^2+^ mishandling has been increasingly linked to various neuropsychiatric and neurodegenerative disorders [22]. Together, these proteins provide insight into the molecular changes associated with stress, neuroplasticity, and antidepressant effects in brain development [23].

It was indeed reported in our previous study that the maternal CUS enhanced the excitability of 5-HT neurons in offspring, and perinatal bupropion potentiated the stimulatory effect of the CUS on 5-HT neuronal firing activity in offspring [19]. The present study aims to test the hypothesis that the offspring behavioral changes induced by perinatal mirtazapine, administered by its own or in combination with the CUS, are mediated via the altered hippocampus Cornu Ammonis-1/3 (CA1/3) glutamate, dorsal raphe nucleus (DRN) 5-HT, and ventral tegmental area (VTA) dopamine neuronal firing activity.

The present study aimed to investigate the effects of the maternal pregestational CUS, perinatal mirtazapine treatment, and their combination, on the anxious behavior of the adult male and female offspring, expression of the pro-neuroplastic proteins in their brains, excitability of brainstem monoamine and hippocampal glutamate neurons, as well as expression and activity of RyR2 channels.

## Methods

### Animals

Female nulliparous Wistar rats, weighing 200–220 g, were obtained from the Department of Toxicology and Laboratory Animal Breeding, Institute of Experimental Pharmacology and Toxicology, Centre of Experimental Medicine of the Slovak Academy of Sciences, Dobra Voda, Slovakia.

### Pre-gestational stress

Female nulliparous Wistar rats were allowed to acclimatize for at least one week, and then randomly divided into the CUS or non-CUS groups, as previously described [16–19]. For the detailed protocol of the CUS administration, see Supplementary Materials.

### Mating, perinatal antidepressant treatment, and subsequent manipulations

One week after the end of the CUS procedure, females were mated with males. Mirtazapine (10 mg/kg/day, per oral) was administered from day 10 of gestation until delivery. For the detailed protocol of mirtazapine administration, see Supplementary Materials. The offspring were weaned on postpartum day 21 and housed in litter groups of four animals per cage of the same sex. All experiments were carried out on male and female offspring of antidepressant or vehicle treated CUS or non-CUS dams who had reached the age of 48–56 days. As in our previous studies [16–19], electrophysiological and proteomic experiments were carried out on different animals, at least 24 h after the performance of behavioral experiments.

### EPM test

The anxiety behavior of the adult offspring of the stressed and non-stressed dams treated with mirtazapine or vehicle during the gestation was measured using EMP. For the detailed protocol, see the Supplementary Materials.

### Assessment of the expression of pro-neuroplasticity proteins

Brain samples were extracted and whole hippocampi were excised for quantitative protein Western blot analysis. For the detailed protocol of the Western blot analysis of NDNF, PSD95, GFAP, and GLUR levels, see the Supplementary Materials.

### Quantification of the optical densities of synaptophysin

Sections of the dorsal hippocampus were analyzed for optical densities of synaptophysin. For the detailed protocol, see Supplementary Materials.

### Electrophysiology in vivo

The spontaneous firing activity of the hippocampal glutamate, DRN 5-HT, and VTA dopamine neurons were performed using extracellular single-unit in vivo electrophysiology. For the detailed protocol, see Supplementary Materials.

### Expression and activity of RyR2

The brain endoplasmic reticulum (ER) microsomes enriched in RyR2 channels were isolated from rat subcortical structures located beneath the cerebral cortex, following isolation protocol described by Bilmen and Michelangeli [24]. For details, see see Supplementary Materials.

### Data analysis

Action potentials (spikes) were detected using the spike sorting algorithm, with version 6.02 of Spike2 software (Cambridge Electronic Design, Cambridge, UK). The neuronal firing rate and burst activity characteristics were calculated using the burstiDAtor software (www.github.com/nno/burstidator), in accordance with our previous publications [19, 25–27]. Statistical assessments were carried out using SigmaPlot 12.5 software (Systat Software Inc, Chicago, IL, USA). A three-way analysis of variance (ANOVA), with the factors of sex, maternal CUS, and perinatal mirtazapine, followed by a Bonferroni *post-hoc* test, was used to evaluate the impacts of maternal CUS and antidepressant treatment and the sex of the offspring, on the offspring behaviour, expression of pro-neuroplastic proteins in the offspring brain, synaptophysin optical density, firing activity characteristics of the hippocampal glutamate and brainstem monoamine neurons, and opening of the RyR2. When three-way ANOVA, due to substantial sex differences, did not detect significant effects of maternal CUS and/or perinatal mirtazapine, two-way ANOVA with the maternal CUS and mirtazapine as factors of comparison was conducted separately for male and female offspring. Before the analysis by ANOVA, data normality and homoscedasticity were verified using Shapiro-Wilk’s and Levene’s tests, respectively. A probability of p ≤ 0.05 was considered significant.

## Results

### Effects of the pre-gestational maternal CUS, perinatal mirtazapine, and their combination on the offspring behavior during the EPM test

**Fig. 1:**
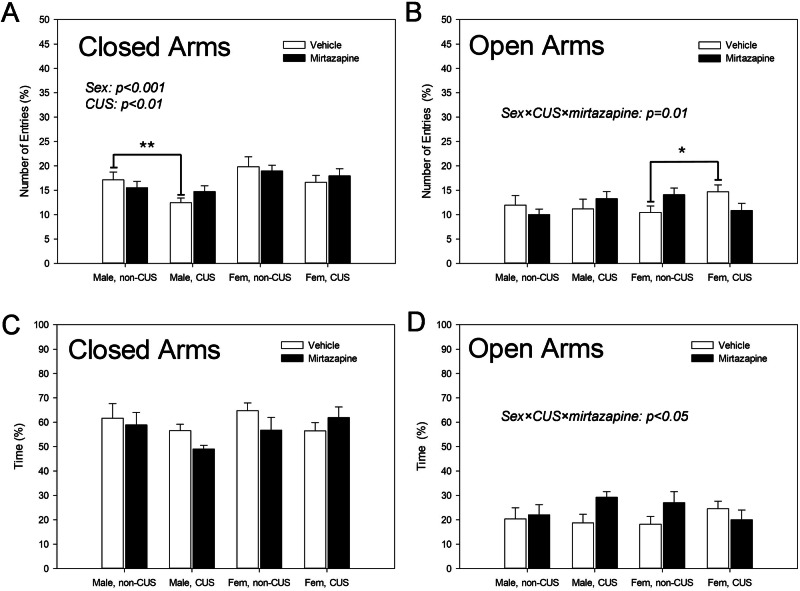
Effects of maternal stress and mirtazapine on the offspring behavior. Number of entries to the closed (**A**) and open (**B**) arms and time spent in the closed (**C**) and open (**D**) arms of the elevated plus maze (EPM) apparatus for male and female (fem) offspring of the dams experienced (CUS) or not experienced (non-CUS) pregestational chronic unpredictable stress and treated with mirtazapine or vehicle during the gestation. *p < 0.05 and p < 0.01, between-group comparison, Bonferroni post-hoc tests.

There were significant effects of sex (F_1,76_ = 11.54, p = 0.001) and maternal CUS (F_1,76_ = 5.87, p = 0.02, three-way ANOVA) on the number of entries to the closed arms of the EPM apparatus in the offspring (Fig. 1A). There was no effect of maternal mirtazapine and no interactions between the factors of the comparison. Bonferroni *post-hoc* test did not detect any significant difference between any specific groups of animals as well.

When two-way ANOVA with the maternal CUS and mirtazapine as factors of comparison was separately applied on the male offspring, significant effect of maternal stress was detected (F_1,37_ = 4.89, p = 0.03). Bonferroni post-hoc test confirmed the decreasing effect of maternal stress (p = 0.01) in the offspring of the vehicle-, but not mirtazapine-treated dams.

With respect to the number of entries to open arms, there was no significant effect of any of the factors of the comparison (Fig. 1B). There was, however, significant sex × maternal CUS × maternal mirtazapine interaction (F_1,78_ = 6.73, p = 0.01, three-way ANOVA). Bonferroni *post-hoc* test did not detect any significant difference between any specific groups of animals as well.

When two-way ANOVA with the maternal CUS and mirtazapine as factors of comparison was separately applied on the female offspring, significant interaction the factors was detected (F_1,37_ = 4.89, p = 0.03). Bonferroni post-hoc test confirmed the increasing effect of maternal stress (p = 0.04) in the offspring of the vehicle-, but not mirtazapine-treated dams.

There were no effects of sex or maternal stress and mirtazapine and no interactions between these factors on the time spent in the closed arms of the EPM apparatus (Fig. 1C).

With respect to the time spent in the open arms (Fig. 1D), there was no significant effect of any of the factors of the comparison. There was, however, significant offspring sex × maternal CUS × maternal mirtazapine interaction (F_1,78_ = 4.33, p = 0.04, three-way ANOVA). Bonferroni *post-hoc* test did not detect any significant difference between any specific groups of animals as well.

### Effects of the pre-gestational maternal CUS, perinatal mirtazapine, and their combination on the expression of pro-neuroplastic proteins

**Fig. 2:**
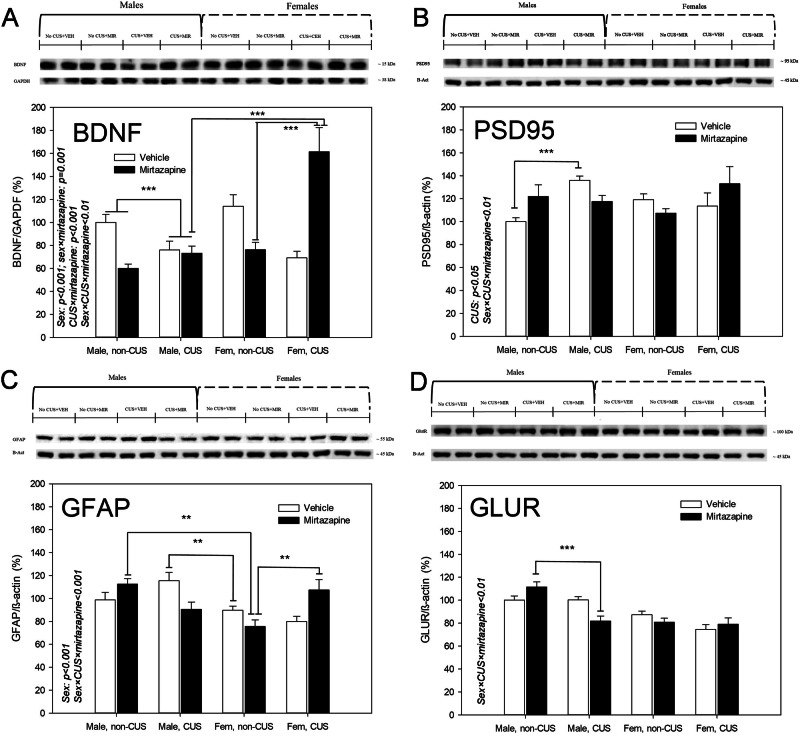
Effects of maternal stress and mirtazapine on the offspring pro-neuroplastic proteins expression. Protein levels of the brain-derived neurotrophic factor (BDNF; **A**), postsynaptic density protein 95 (PSD95; **B**), glial fibrillary acidic protein (GFAP; **C**) and glutamate receptor (GLUR; **D**) in the hippocampus of male and female (fem) offspring of the dams experienced (CUS) or not experienced (non-CUS) pregestational chronic unpredictable stress and treated with mirtazapine or vehicle during the gestation. **p < 0.01 and ***p < 0.001, between-group comparison, Bonferroni post-hoc tests.

With respect to the BDNF protein levels (Fig. 2A), there was a significant effect of the offspring sex (F_1,45_ = 15.50, p < 0.001), offspring sex × maternal mirtazapine (F1,45 = 11.74, p = 0.001), maternal stress × maternal mirtazapine (F_1,45_ = 34.89, p < 0.001), and offspring sex × maternal stress × maternal mirtazapine interactions (F_1,45_ = 10.76, p = 0.002, three-way ANOVA). Bonferroni *post-hoc* test detected suppressing effect of the maternal CUS on BDNF level in male offspring (p < 0.001, regardless maternal mirtazapine treatment), significant difference between male and female offspring of the dams exposed to both CUS and mirtazapine (p < 0.001, with the value higher in females), and a robust increasing effect of the maternal CUS and in female offspring of mirtazapine-treated dams (p < 0.001).

Regarding the PSD95 (Fig. 2B), there was a significant effect of the maternal stress (F_1,49_ = 6.05, p = 0.02) and significant interaction between the offspring sex, maternal stress, and maternal mirtazapine treatment (F_1,49_ = 11.49, p = 0.002, three-way ANOVA). Bonferroni *post-hoc* test also unveiled a significant increasing effect of maternal stress in male offspring of the vehicle-, but not mirtazapine-treated dams (p = 0.02).

With respect to the GFAP (Fig. 2C), there was a significant effect of the offspring sex (F_1,46_ = 13.28, p < 0.001) and significant offspring sex × maternal stress × maternal mirtazapine interaction (F_1,46_ = 18.75, p < 0.001, three-way ANOVA). Bonferroni *post-hoc* test detected sex differences in the offspring of non-stressed dams treated with mirtazapine and stressed dams treated with vehicle (p < 0.002, and the value in males higher than in females in both cases), as well as an increasing effect of the maternal stress and female offspring of mirtazapine-treated dams (p < 0.002).

Regarding the GLUR (Fig. 2D), there was a significant offspring sex × maternal stress × maternal mirtazapine interaction (F_1,49_ = 11.49, p < 0.002, three-way ANOVA). Bonferroni *post-hoc* test unveiled suppressing effect of the maternal CUS in male offspring of mirtazapine-treated dams (p < 0.001).

### Effects of the pre-gestational maternal CUS, perinatal mirtazapine, and their combination on the synaptophysin optical density in the hippocampus

**Fig. 3:**
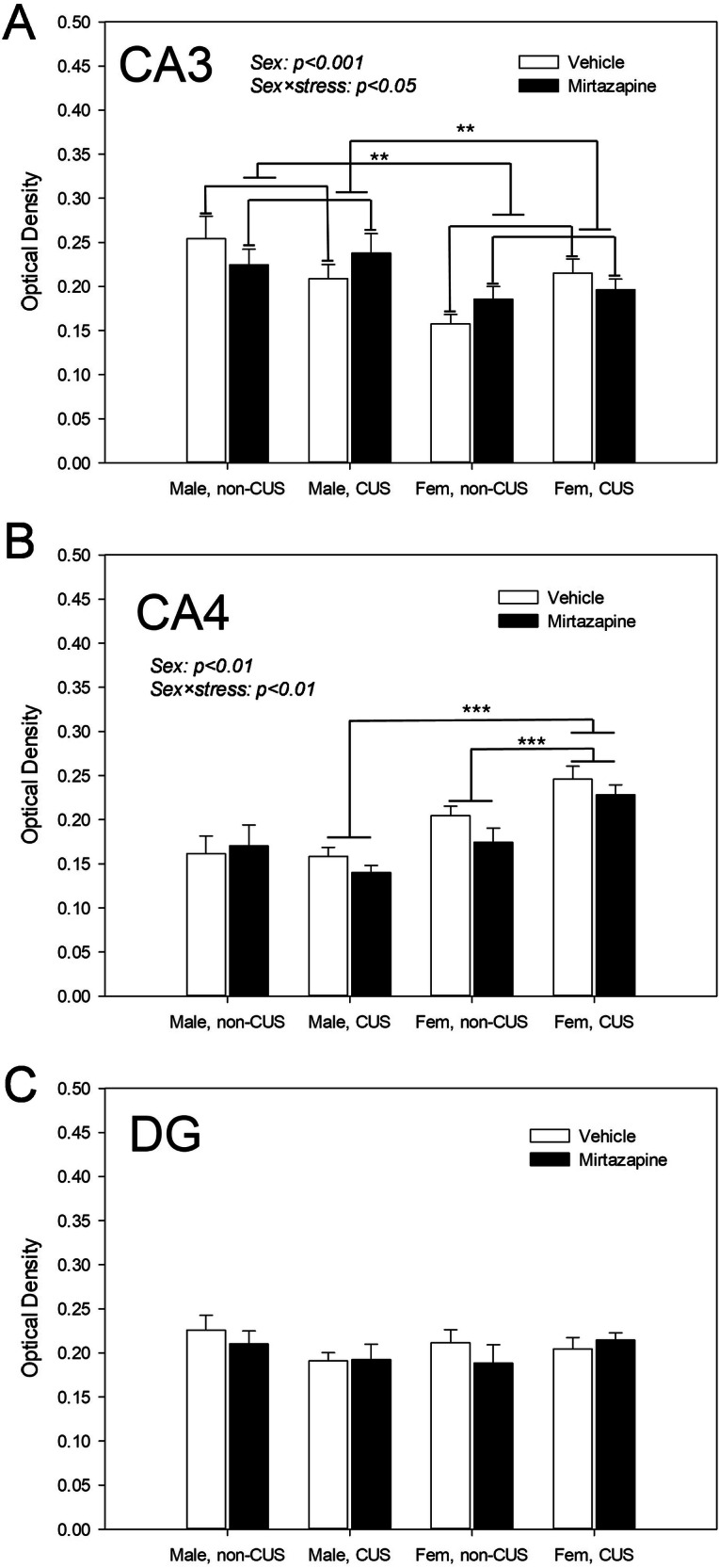
Effects of maternal stress and mirtazapine on the offspring hippocampal synaptophysin levels. Synaptophysin optical density in the cornu Ammonis 3 (CA3; **A**), cornu Ammonis 4 (CA4; **B**), and dentate gyrus (DG; **C**) of male and female (fem) offspring. **p < 0.01 and ***p < 0.001, between-group comparison, Bonferroni post-hoc tests.

Figure 3 illustrates the synaptophysin optical density in the hippocampal *Cornu Ammonis* areas CA3 (A) and CA4 (B) and in the dentate gyrus (C). In the CA3, there was significant effect of the offspring sex (F_1,58_ = 12.03, p = 0.001) and significant interactions between offspring sex and maternal CUS (F_1,58_ = 4.16, p = 0.05), and maternal stress and CUS (F_1,58_ = 4.61, p = 0.04). Bonferroni *post-hoc* test reported sex differences in the offspring of vehicle- (p = 0.01) and mirtazapine-treated (p = 0.02) rats, regardless of maternal CUS exposure (values in females lower compared to t males in both cases).

With respect to the CA4, there was significant effect of the offspring sex (F_1,59_ = 30.06, p < 0.001) and significant offspring sex × maternal CUS interaction (F_1,59_ = 9.61, p = 0.003). Bonferroni *post-hoc* test detected effect of the maternal CUS on CA4 synaptophysin in females (p < 0.001), but not in males. There was a statistically significant difference between the sexes in the offspring of the stressed (p < 0.001), but not in non-stressed dams. CA4 synaptophysin levels were higher in females compared to males.

In the dentate gyrus, neither the difference between the sexes nor the effect of the maternal CUS or mirtazapine, or any interaction between the factors of comparison, were observed.

### Effects of the pre-gestational CUS, perinatal mirtazapine, and their combination on the firing activity of glutamate neurons in the hippocampus of male and female offspring

**Fig. 4:**
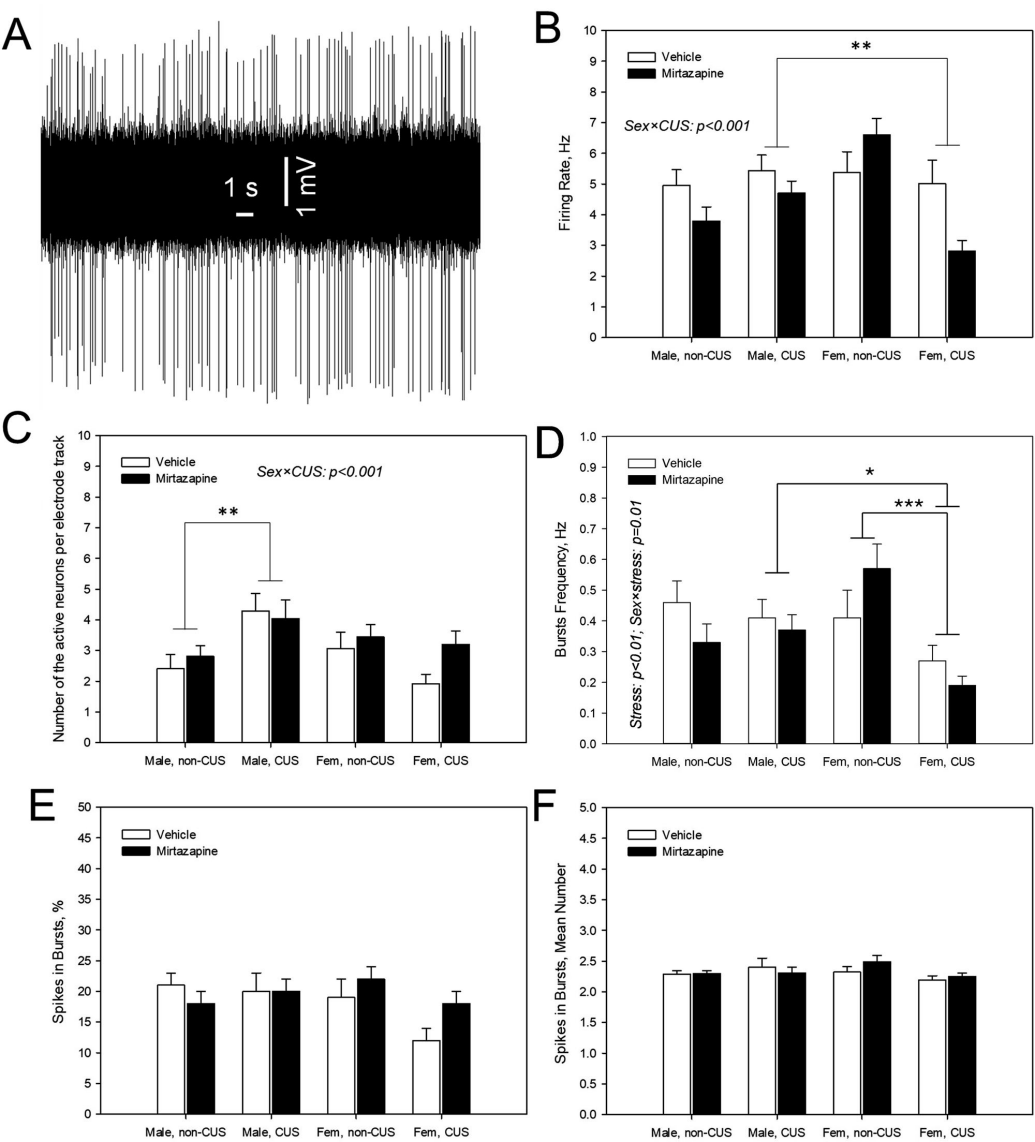
Effects of maternal stress and mirtazapine on the offspring hippocampal glutamate neuronal firing activity. Representative recording from a hippocampal glutamate neuron (**A**; male offspring of the vehicle-treated not stressed dam) and mean spontaneous firing rate (**B**), number of spontaneously active neurons per electrode descend (**C**), burst frequency (**D**), percent of spikes occurring in the bursts (**E**), and number of spikes per burst (**F**) of the hippocampal glutamate neurons of male and female (fem) offspring of the dams experienced (CUS) or not experienced (non-CUS) pregestational chronic unpredictable stress and treated with mirtazapine or vehicle during the gestation. *p < 0.05, **p < 0.01 and ***p < 0.001, between-group comparison, Bonferroni post-hoc tests.

Representative recording from a hippocampal glutamate neuron is shown on Fig. 4A. With respect to the mean spontaneous firing rate of the neurons (Fig. 4B), there was a significant interaction between the offspring sex and maternal CUS (F_1,490_ = 12.90, p < 0.001, three-way ANOVA). Bonferroni post-hoc test detected a sex difference in the offspring of the stressed dams (p < 0.003, with the value higher in males).

Regarding the average number of the spontaneously active neurons per electrode descend (Fig. 4C), three-way ANOVA revealed significant offspring sex × maternal stress interaction (F_1,150_ = 8.47, p < 0.004, three-way ANOVA). Bonferroni post-hoc test detected sex differences in the offspring of the stressed dams (p = 0.002, with the value in males higher than in females) and emphasized the increasing effect of the maternal CUS in male (p = 0.004, regardless maternal mirtazapine treatment), but not in female offspring.

With respect to the bursts’ frequency (Fig. 4D), there was a significant effect of the maternal stress (F_1,150_ = 6.83, p = 0.009) and significant interaction between offspring sex and maternal stress (F_1,150_ = 6.78, p = 0.01, three-way ANOVA). Bonferroni post-hoc test showed significant sex differences in the offspring of the stressed dams (p < 0.03, with the value in males higher than in females) and suppressing effect of the maternal stress in female (p < 0.001), but not male offspring.

Other characteristics of the burst firing, namely, percent of the spikes occurring in the bursts (Fig. 4E) and the mean number of spikes in bursts (Fig. 4F), were not affected by the maternal stress or mirtazapine treatment, neither they had sex differences.

### Effects of the pre-gestational CUS, perinatal mirtazapine, and their combination on the firing activity of 5-HT neurons in the DRN of male and female offspring

**Fig. 5:**
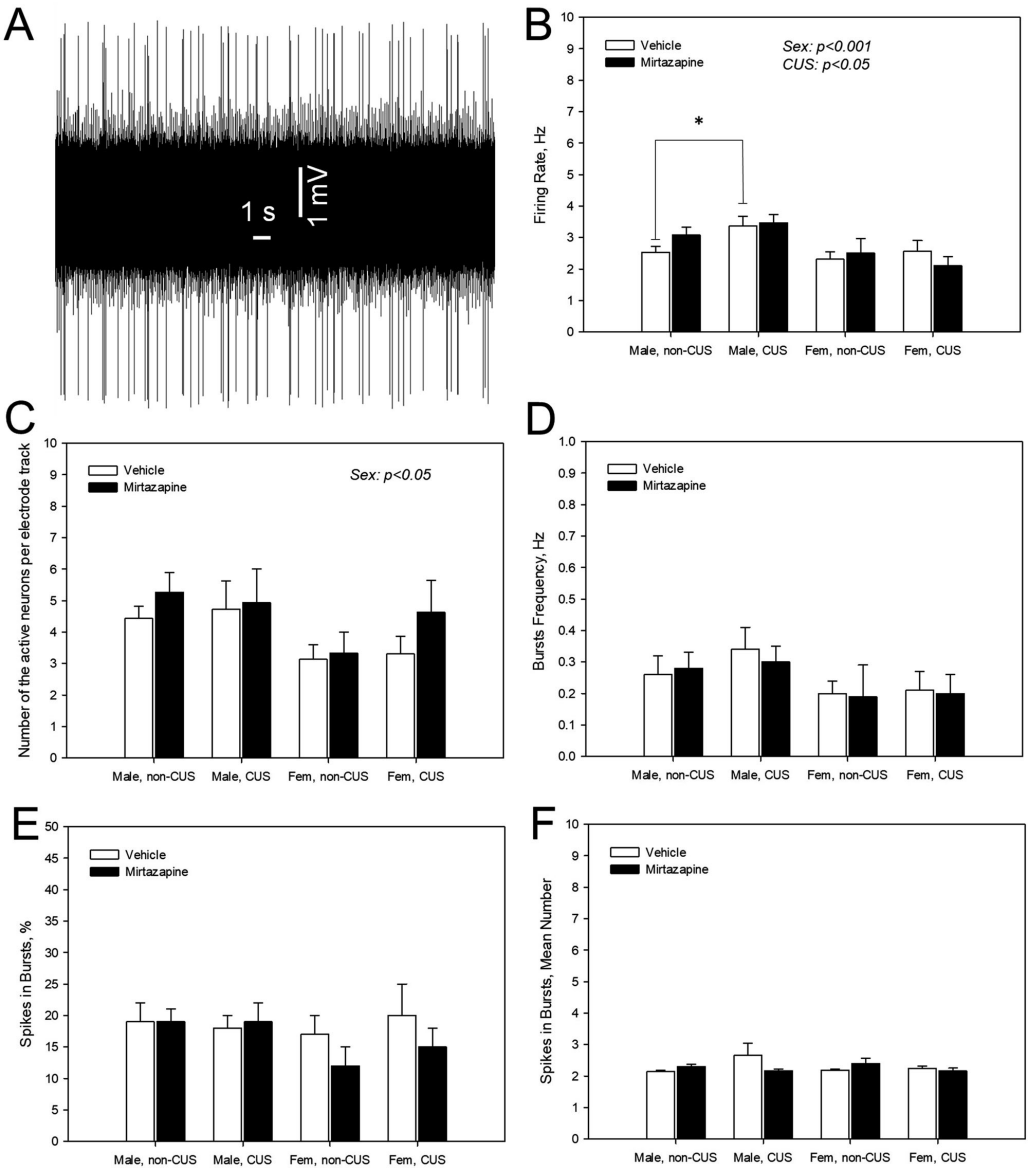
Effects of maternal stress and mirtazapine on the offspring serotonergic neuronal firing activity. Representative recording from a dorsal raphe nucleus (DRN) serotonin (5-HT) neuron (**A**; male offspring of the vehicle-treated not stressed dam) and mean spontaneous firing rate (**B**), number of spontaneously active neurons per electrode descend (**C**), burst frequency (**D**), percent of spikes occurring in the bursts (**E**), and number of spikes per burst (**F**) of the DRN 5-HT neurons of male and female (fem) offspring of the dams experienced (CUS) or not experienced (non-CUS) pregestational chronic unpredictable stress and treated with mirtazapine or vehicle during the gestation. *p < 0.05, between-group comparison, Bonferroni post-hoc tests.

Representative recording from a DRN 5-HT neuron is shown on Fig. 5A. With respect to the mean spontaneous firing rate of the neurons (Fig. 5B), three-way ANOVA revealed the significant effect of the offspring sex (F_1,400_ = 10.99, p < 0.001); the values in females lower comparing to the males. When two-way ANOVA (using the maternal stress and maternal mirtazapine treatment as factors of comparison) was applied on the male offspring only, it revealed significant effect of the maternal stress (F_1,263_ = 5.22, p = 0.02). Maternal stress increased the firing rate of 5-HT neurons in the male offspring of the vehicle-, but not mirtazapine-treated rats (p = 0.04, Bonferroni post-hoc test).

Regarding the average number of the density of the spontaneously active neurons (Fig. 5C), only a sex difference (F_1,93_ = 4.84, p < 0.04, three-way ANOVA) was unveiled. The values in females were lower compared to the males.

With respect to the parameters of the burst firing, such as bursts’ frequency (Fig. 5D), percent of the spikes occurring in the bursts (Fig. 5E) and the mean number of spikes in bursts (Fig. 5F), they were not affected by the offspring sex, maternal stress nor mirtazapine treatment.

### Effects of the pre-gestational CUS, perinatal mirtazapine, and their combination on the firing activity of dopamine neurons in the VTA of male and female offspring

**Fig. 6:**
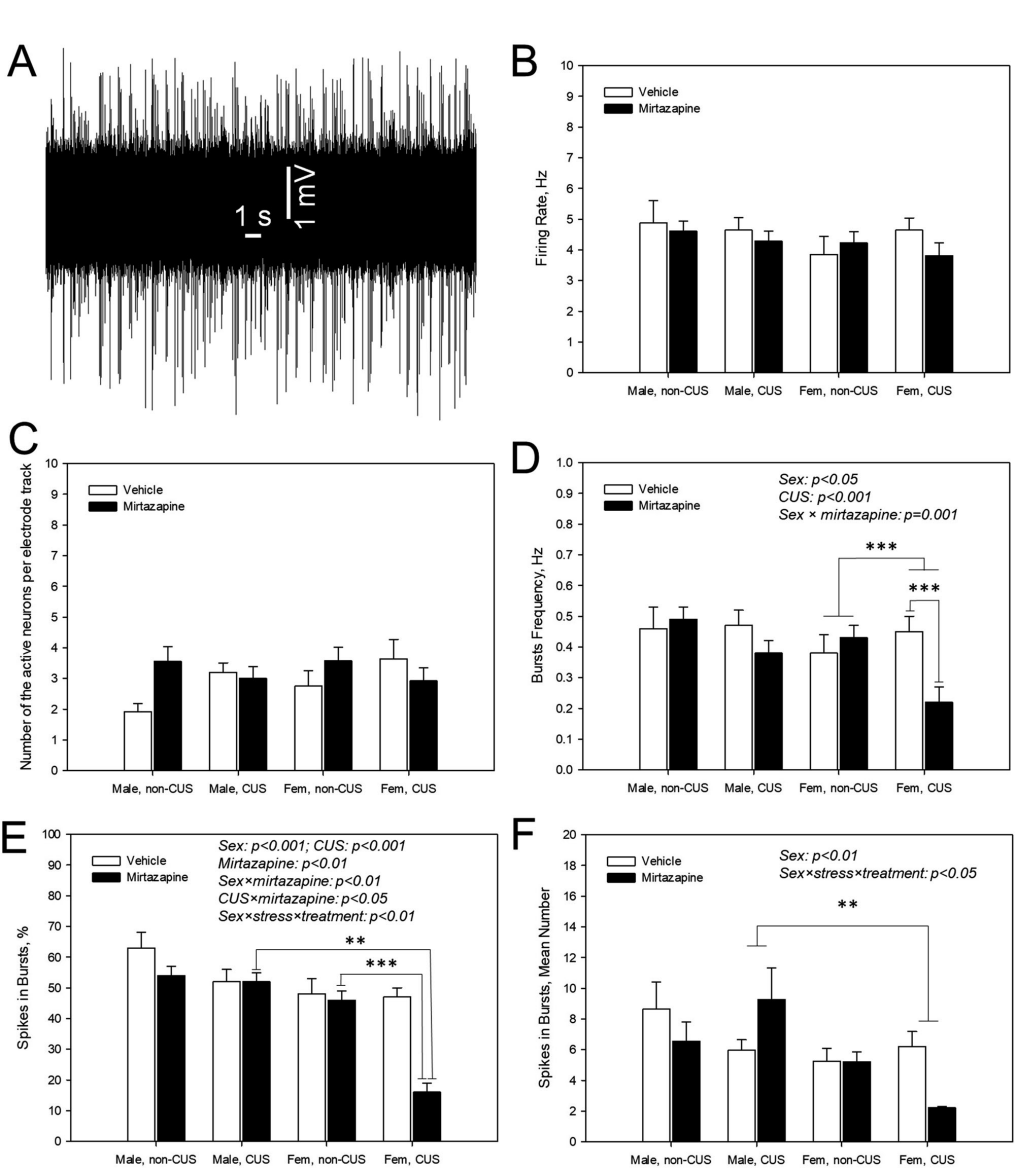
Effects of maternal stress and mirtazapine on the offspring dopaminergic neuronal firing activity. Representative recording from a ventral tegmental area (VTA) dopamine neuron (**A**; male offspring of the vehicle-treated not stressed dam) and mean spontaneous firing rate (**B**), number of spontaneously active neurons per electrode descend (**C**), burst frequency (**D**), percent of spikes occurring in the bursts (**E**), and number of spikes per burst (**F**) of the VTA dopamine neurons of male and female (fem) offspring of the dams experienced (CUS) or not experienced (non-CUS) pregestational chronic unpredictable stress and treated with mirtazapine or vehicle during the gestation. **p < 0.01 and ***p < 0.001, between-group comparison, Bonferroni post-hoc tests.

Representative recording from a DRN 5-HT neuron is shown on Fig. 6A. Offspring sex, maternal stress or mirtazapine treatment did not affect the firing rate (Fig. 6B) and the average number of the spontaneously active neurons per electrode track (Fig. 6C).

With respect to the bursts’ frequency (Fig. 6D), significant effects of the offspring sex (F_1,346_ = 7.48, p = 0.007) and maternal stress (F_1,346_ = 6.83, p = 0.009) and significant interaction between maternal sex and maternal mirtazapine treatment (F_1,346_ = 10.59, p = 0.001, three-way ANOVA) were observed. Bonferroni pos-hoc test revealed the suppressing effects of the maternal CUS in female offspring (p = 0.001) and maternal mirtazapine in female offspring of stressed dams (p = 0.001).

Regarding the percentage of the spikes occurring in the bursts (Fig. 6E), there were significant effects of the offspring sex (F_1,346_ = 36.49, p < 0.001), maternal stress (F_1,346_ = 19.19, p < 0.001) and mirtazapine treatment (F_1,346_ = 6.99, p < 0.009), as well as significant offspring sex × maternal mirtazapine (F_1,346_ = 8.20, p = 0.004), maternal stress × maternal mirtazapine (F_1,346_ = 6.28, p < 0.02), and offspring sex × maternal stress × maternal mirtazapine (F_1,346_ = 6.16, p = 0.01) interactions. Bonferroni post-hoc test detected a sex difference in the offspring of the mirtazapine-treated non-stressed (p = 0.007) and stressed dams (p < 0.001, the value in females lower compared to the males in both cases) and suppressing effect of maternal stress in female offspring of mirtazapine-treated dams (p < 0.001).

With respect to the mean number of spikes in burst (Fig. 6F), there was a significant effect of the offspring sex (F_1,346_ = 7.49, p < 0.007) and significant offspring sex × maternal stress × maternal mirtazapine interaction (F_1,346_ = 5.34, p < 0.02). Bonferroni post-hoc test revealed a sex difference in the offspring of the stressed dams, regardless of mirtazapine treatment (p = 0.004), with the value in females lower than in males.

### Effects of the pre-gestational CUS, perinatal mirtazapine, and their combination on the expression and activity of RyR2 channels

Expression of RyR2 protein and opening of RyR2 channels were not affected by the offspring sex or by maternal stress or mirtazapine (see Supplementary Materials, Fig. S1).

## Discussion

We found that pregestational CUS decreased number of entries to the closed arms in male offspring and increased number of entries to the open arms in female offspring. This anxiolytic like effect was observed in the offspring of the vehicle-, but not mirtazapine-treated dams. Maternal CUS increased PSD95 expression in male offspring, increased synaptophysin in females, and decreased BDNF in both sexes. In females, pregestational CUS-induced decrease in BDNF expression was reversed by perinatal mirtazapine. Combination of pre-gestational CUS and perinatal mirtazapine increased the GFAP expression in the females and decreased GLUR expression in the males. Pre-gestational CUS decreased the excitability of the hippocampal glutamate and VTA dopamine neurons; mirtazapine enhanced this effect. With respect to 5-HT neurons, maternal CUS increased their firing activity. The key results of our study are summarized in Table 1.

**Table 1:** Effects of maternal stress and mirtazapine on the offspring. Effects of the maternal chronic unpredictable stress, perinatal mirtazapine, and their combination, on the behavioral, neurochemical, and neurophysiological characteristics of the male (A) and female (B) offspring; ↑, increasing effect; ↓, decreasing effect; 0/↑ perinatal mirtazapine might have an increasing effect on these characteristics, even though this effect was not statistically significant by its own.

Characteristic	Maternal CUS	Mirtazapine	Combination
***A: Male offspring***			
Anxiety	↓	0/↓	
BDNF expression			↓
PSD95 expression	↑		
GFAP expression			
GLUR expression			↓
Synaptophysin optical density	↓		
Hippocampal glutamate neuronal activity			↑
DRN 5-HT neuronal activity	↑	0/↑	
VTA dopamine neuronal activity			
***B: Female offspring***			
Anxiety	↓	0/↓	
BDNF expression			↑
PSD95 expression			
GFAP expression			↑
GLUR expression			
Synaptophysin optical density			↑
Hippocampal glutamate neuronal activity			↓
DRN 5-HT neuronal activity			
VTA dopamine neuronal activity			↓

Our findings indicate that maternal CUS influenced specific aspects of EPM behavior in the offspring. In males, it reduced the number of entries into the closed arms, while in females, it increased entries into the open arms. It can be summarized that pregestational CUS had a slight anxiolytic effect on the offspring. This finding is surprising, since maternal stress is usually associated with increased anxiety in offspring. It was reported that the maternal immune activation (MIA) during the gestation had anxiogenic effect on the offspring, as measured by the EPM [28] and novelty-induced hyperphagia tests [29]. It is this possible that the exact effect of the maternal exposure to the stressors on the offspring behaviour depends on the nature of the stressor (CUS *versus* MIA) and/or on the timing of the exposure (pregestational *versus* prenatal). While prenatal MIA increased the offspring anxiety, putatively, *via* the mechanism involving decreased 5-HT neurotransmission [25], pregestational CUS might induce compensatory mechanism(s) in the maternal and/or offspring organism, resulting in decreased anxiety. Notably, the anxiolytic effect of the maternal CUS was observed only in the offspring of vehicle-treated rats, not those treated with mirtazapine. Perinatal mirtazapine might thus also have slight anxiolytic effects, even though it did not reach the level of statistical significance.

It was found that pregestational CUS decreased BDNF expression in the offspring brain. In females, but not in males, perinatal mirtazapine reversed the suppressing effect of the pre-gestational CUS on BDNF expression in the offspring brain. With respect to PSD95, maternal CUS increased its expression in the brain of the male, but not female offspring. Perinatal mirtazapine might also have similar effect on PSD95 expression, even though it did not reach the level of statistical significance on its own. Thus, significant difference between the offspring of the stressed and non-stressed dams was observed only in animals perinatally exposed to the vehicle, but not to mirtazapine. Combination of pre-gestational CUS and perinatal mirtazapine, but none of these factors by their own, increased the GFAP expression in the female and decreased GLUR expression in the male offspring brain.

CUS may diminish BDNF expression in female offspring but not in male offspring due to sex-specific differences in stress response and brain plasticity. These differences are largely driven by hormonal influences, particularly the interaction of sex hormones like estrogen and testosterone with neurotrophic factors such as BDNF [30]. In females, estrogen plays a critical role in regulating BDNF expression, especially in brain areas like the hippocampus, which is sensitive to stress [31]. Chronic stress can disrupt the estrogen-BDNF pathway, leading to reduced BDNF levels. Males, on the other hand, have different hormonal responses and may be more resilient to the BDNF-lowering effects of stress due to testosterone or differential activation of stress-related brain circuits [32]. Additionally, stress may trigger different neuroinflammatory responses or epigenetic changes in males and females, contributing to the sex-specific regulation of BDNF expression [33].

The interaction of mirtazapine with chronic stress produces sex-specific neurobiological outcomes-enhancing GFAP and astrocyte activation in females, potentially exacerbating anxiety-related behaviors, while reducing glutamate receptor levels and improving stress resilience in males, leading to anxiolytic effects. These sex differences highlight the complex interplay between stress, neuroinflammation, and neurotransmission in shaping behavioral responses.

It was found that the maternal CUS increased synaptophysin optical density in the offspring hippocampus, especially, in its CA3 area. This effect was observed in female, but not in male offspring. Perinatal mirtazapine did not affect offspring hippocampal synaptophysin; neither did pregnancy with the effect of pregestational CUS.

The increase in synaptophysin optical density in female but not male offspring may be due to sex-specific hormonal regulation, differential stress responses, and possible compensatory plasticity mechanisms in the female brain, which may help offset the adverse effects of maternal chronic stress.

Chronic stress during pregnancy can induce epigenetic changes that influence brain development in offspring. These changes might alter the expression of synaptic proteins like synaptophysin in a sex-dependent manner [34]. In females, maternal stress might induce compensatory mechanisms, such as increased synaptic plasticity, as a way to cope with the altered brain environment, leading to higher synaptophysin levels [35]. In addition, estrogen may interact with synaptic proteins like synaptophysin more prominently in female offspring. This hormone may enhance synaptic resilience to stress, potentially increasing synaptophysin expression in females but not in males, who rely more on testosterone, which might have different effects on synaptic function [36].

We found that maternal CUS decreased the density of the spontaneously active hippocampal neurons, their mean firing rate, and the burst mode of their firing. This effect was sex-specific and observed in female, but not in male rats. It is known that the burst firing of glutamate neurons enhances the nerve terminal neurotransmitter release, in comparison with the same amount of action potentials fired in a single-spike mode [37]. Pregestational stress is therefore likely to decrease hippocampal glutamate transmission in female offspring. It is thus possible that the decrease in hippocampal glutamate transmission, observed in females, but not in males, is responsible, at least in part, for the anxiogenic effect of pregestational maternal stress, that was also observed in female, but not in male offspring. Interestingly, similar decreasing effect of maternal stress on the firing activity was also observed in cultivated neurons that are isolated from the hippocampi of newborn offspring of the dams experienced pregestational CUS [38] or prenatal immune activation [39].

The link between reduced hippocampal glutamate transmission and anxiety has been shown in previous studies. Widman and coauthors demonstrated that the low novelty response (LR) rats, a genetic breed characterized by high anxiety, had decreased hippocampal long-term potentiation (LTP) and reduced density of the dendritic spines on hippocampal pyramidal neurons [40]. On the other hand, high levels of anxiety in rats experienced social defeat stress [41] and in mice lacking serotonin-1A (5-HT_1A_) receptor [42] were associated with increased LTP and excitatory post-synaptic currents (EPSC), respectively. In our previous study, an anxiolytic effect of a selective delta-opioid receptor (DOR) agonist SNC80 was accompanied by an elevated firing rate and increased burst activity of hippocampal neurons [43].

It was found that the mean firing rate of the spontaneously active 5-HT neurons, as well as their density, were higher in the males comparing to the females. Similar sex-related differences in the firing activity of 5-HT neurons were observed in the previous studies from our [19] and other laboratories [44].

Similarly to our previous study [19], we found that pregestational CUS increased the basal firing rate of 5-HT neurons in male, but not in female offspring. Since the firing activity of the DRN 5-HT neurons determines limbic 5-HT neurotransmission [45], pregestational CUS might increase 5-HT tone in the male offspring. As it was already suggested [19], maternal CUS-induced increase in limbic 5-HT transmission in the male offspring might be a compensatory mechanism designated to prevent the negative effect of the maternal stress on the offering brain.

Perinatal mirtazapine might also have an increasing effect on the firing activity of 5-HT neurons in male rats, even though this effect was not statistically significant by its own. Thus, statistically significant difference between the offspring of the stressed and non-stressed dams was observed only in animals perinatally exposed to the vehicle, but not to mirtazapine. This observation is consistent with the stimulatory effect of the sustained mirtazapine on 5-HT neurons reported in previous studies [46, 47].

It is well established that 5-HT neurotransmission is fundamental in anxiety [48]. Maternal and CUS and mirtazapine-induced stimulation of the offspring 5-HT neurons might be therefore consistent with the anxiolytic effects of these maternal factors on the offspring behavior during the EPM test. Notably, the anxiogenic effect of another maternal stressor, MIA, was accompanied with decreased firing activity of the DRN 5-HT neurons [25]. The presence of this compensatory mechanism in male, but not in female rats might explain the fact that the anxiolytic effect of the maternal stress and mirtazapine was observed in male, but not in female offspring.

We found that pregestational CUS led to the decreased burst firing of dopamine neurons in female, but not in male offsprings’ VTA. Perinatal mirtazapine robustly potentiated the effect of pregestational CUS on the burst firing of dopamine neurons in female offspring’ VTA. Burst firing of dopamine neurons is associated with higher efficiency of neurotransmitter release from the nerve terminals [49]. Maternal CUS and mirtazapine may thus suppress dopamine neurotransmission in female offspring. Maternal CUS and mirtazapine-induced suppression of dopamine neurotransmission might be thus involved in the anxiogenic effect of these factors, that was also observed in female, but not male offspring.

The involvement of the mesolimbic dopamine transmission in anxiety was indeed reported in previous studies. It was stated that postpartum anxiety and depressive-like behavior in rats were associated with attenuated population activity in the VTA [50]. On the other hand, increased anxiety in a rat model of neuropathic pain was associated with elevated accumulation of dopamine levels [51]. Finally, we had recently described that the anxiolytic effect of DOR agonist SNC80 was accompanied by elevated firing rate and increased burst activity of the VTA dopamine neurons [43].

Ca^2+^ signaling in the brain plays a crucial role in neuronal viability, synaptic plasticity, and higher cognitive processes [20, 21, 52, 53], suggesting that Ca^2+^ mishandling contributes to various neuropsychiatric and neurodegenerative disorders [22, 54–59]. RyR2, a key intracellular Ca^2+^ channel, is significantly implicated in neuronal Ca^2+^ signaling and several studies suggest that phosphorylation-induced increase in RyR activity may contribute to AD pathogenesis [56, 59–62].

Implication of RyR2 channels in pathogenesis of neuropsychiatric disorders has started to emerge only recently [63, 64]. However, the primary efforts have focused on identifying mutations in the RYR genes that encode three distinct isoforms, rather than directly investigating potential RyR dysfunction and post-translational modifications. We found that neither amount of the RyR2 channel (a dominant isoform in the brain [65, 66]), its resting activity, or phosphorylation state was altered. Thus, we can exclude the potential contribution of this Ca^2+^ channel in changes in neuronal excitability of the offspring caused by the pre-gestational maternal CUS, perinatal mirtazapine, and their combination.

One of the primary limitations of our study is the fact that the offspring behavior was assessed using the EPM test only. In future studies, battery of the tests aiming to characterize different behavioral aspects (e.g., sucrose preference test and forced swim test as markers for anhedonia and depressive-like behavior, respectively) should be applied for the complex assessment of maternal stress and perinatal antidepressant treatment on the offspring.

In conclusion, perinatal mirtazapine did not alter anxious behavior of the offspring by its own. Maternal stress, however, had an anxiolytic effect on the offspring of the vehicle-treated dams, accompanied by a stimulation of 5-HT neurons. Perinatal mirtazapine might also have slight effects in the same direction, even though they are not reaching the level of statistical significance. Stimulatory effect of perinatal mirtazapine on BDNF expression in the offspring of the stressed dams might be linked with increased 5-HT neurotransmission and with decreased anxiety. Maternal stress and mirtazapine had a suppressing effect on the burst firing of the offspring VTA dopamine and hippocampal glutamate neurons; it might be linked with the decreased expression of the GLUR. The effects of maternal stress and mirtazapine on the burst firing of the central neurons in offspring are not mediated via mechanism(s) involving the RyR2 channel. Epigenetic regulation of the other key genes linked with 5-HT and glutamate neurotransmission, such as BDNF and/or GLUR, may have a critical role in the offspring neurophysiological and behavioral changes, triggered by maternal stress and antidepressant treatment. Thus suggestion should be examined in future studies.

## Supplementary information

Supplemental Material

## Data Availability

Data will be made available on request.

## Animal experiments

All experimental procedures were approved by the Animal Health and Animal Welfare Division of the State Veterinary and Food Administration of the Slovak Republic (Permit number Ro 3592/15-221) and confirmed to the Directive 2010/63/EU of the European Parliament and of the Council on the Protection of Animals Used for Scientific Purposes.
