# Supplementary material for: Effects of pre-gestational exposure to the stressors and perinatal mirtazapine administration on the excitability of hippocampal glutamate and brainstem monoaminergic neurons, hippocampal neuroplasticity, and anxiety-like behavior in rats
Source: Mol Psychiatry. 2025 Aug 25;31(2):726–38. doi: 10.1038/s41380-025-03161-3 (PMC12815651; doi:10.1038/s41380-025-03161-3)
Supplement: Supplementary file 1 — Supplemental Material [file 41380_2025_3161_MOESM1_ESM.docx]

**Effects of pre-gestational exposure to the stressors and perinatal mirtazapine administration on the excitability of hippocampal glutamate and brainstem monoaminergic neurons, hippocampal neuroplasticity, and anxiety-like behavior in rats**

By Ruslan Paliokha, Mireia Viñas-Noguera, Stanislava Bukatova, Daniil Grinchii, Jana Gaburjakova, Marta Gaburjakova, Hande Ozbasaka, Roman Dekhtiarenko, Talah Khoury, Lubica Lacinova, Eliyahu Dremencov, and Michal Dubovicky

**Supplementary Materials**

**Detailed methodology**

***Animals***

Female nulliparous Wistar rats, weighing 200-220 g, were obtained from the Department of Toxicology and Laboratory Animal Breeding, Institute of Experimental Pharmacology and Toxicology, Centre of Experimental Medicine of the Slovak Academy of Sciences, Dobra Voda, Slovakia. All experimental procedures were approved by the Animal Health and Animal Welfare Division of the State Veterinary and Food Administration of the Slovak Republic (Permit number Ro 4103/18-221/3) and confirmed to the Directive 2010/63/EU of the European Parliament and of the Council on the Protection of Animals Used for Scientific Purposes.

***Pre-gestational stress***

Female rats were allowed to acclimatize for at least one week, and then randomly divided into the CUS or non-CUS groups, as previously described ^1-4^. The animals were exposed to 1-2 stressors per day. The list of stressors included: (1) overcrowding (24h) – 6 animals housed together in a standard size cage for an extended period; (2) exposure to damp bedding (8h); (3) food deprivation (12h); (4) water deprivation (5h); (5) cage decline (6h) – home cages were tilted in a 45 degree; (6) strobe light during whole day (24h); (7) predator stress – cloth with a cat odor was introduced to the cage vicinity (10h). Control (non-CUS) females, as well as offspring of all females, were kept in the standard cages (20×30×40 cm; 4-5 animals per cage) and under the standard conditions: temperature of 22 ± 2°C, 55 ± 10% humidity, 12 h light/12 h dark cycle with lights on at 7 a.m., and *ad libitum* access to the pelleted food and tap water.

***Mating, perinatal antidepressant treatment, and subsequent manipulations***

One week after the end of the CUS procedure, females were mated with males in a 3:1 ratio. The presence of spermatozoa in vaginal smears was considered day zero of gestation. Mirtazapine (Mikrochem Trade spol., s.r.o.) was diluted in distilled water and orally administered *via* a 1 cm^3^ biscuit from day 10 of gestation until weaning, at 10 mg/kg/day. The dose was chosen based on a body surface area normalization (BSA) conversion used to determine the starting human dose extrapolated from animal studies. The K_m_ factor, which is the ratio of body weight (kg) to body surface area (m^2^), is used to convert doses expressed in mg/kg to units of mg/m^2^. The dams in control groups received 1 cm^3^ biscuits a day filled with vehicle (water). Feeding was completed under the investigator's supervision to ensure the dam consumed the entire biscuit. One day after birth, litters were culled to four males and four females (with eight offspring per cage). The offspring were weaned on postpartum day 21 and housed in litter groups of four animals per cage of the same sex. Dams were randomly divided into four groups: non-CUS + vehicle, non-CUS + bupropion, CUS + vehicle, CUS + bupropion.

***Elevated plus maze (EPM) test***

The offspring anxiety behavior was assessed using the EPM test, as previously described^1-3^. All parts of the EPM apparatus are made of dark polyvinyl plastic. The open and the closed arms of the maze are 50 cm above the floor, 50 cm long, and 10 cm wide. Two tests were running simultaneously. The movements of the rats were tracked with a digital camera, and the individual sessions were analyzed by computer software ANYMAZE^TM^ (Stoelting Europe, Ireland). Mild light was provided by a lamp attached above the open arms of the maze. Each session lasted 5 minutes and was started by placement of the rat in the central area facing the open arms of the maze. After each individual trial, the maze was wiped with a mild detergent. The behaviors scored were time spent in and number of entries to the open and closed arms and their intersection.

***Assessment of the expression of pro-neuroplasticity proteins***

Brain samples were extracted and whole hippocampi were excised for quantitative protein Western blot analysis. After excision, the hippocampi were fixed with liquid nitrogen and stored at -80 °C until use.

The tissue samples from the rat hippocampal tissue were re-suspended in ice cold buffer and homogenized with a glass teflon homogenizer. The homogenates were centrifuged at 800xg for 5 min at 4 °C, the pellets after this centrifugation were discarded and the supernatants were centrifuged again at 16 100xg for 30 min. The supernatants after the second centrifugation called 'soluble fraction' were stored and used for Western blot analysis of cytosolic proteins. Following this centrifugation, the supernatants were discarded again, and the pellets were re-suspended in homogenizing buffer supplemented with 0.2% Triton X-100 and centrifuged at 16 100xg for 5 min. The Triton X-100 soluble supernatants represented the “particulate fraction” used for membrane proteins analysis. Protein concentrations were estimated by the Bradford method.

Samples of soluble protein fractions (BDNF detection) and particulate protein fraction (PSD95, GFAP, GLUR) containing equivalent amounts of proteins per lane were separated by sodium dodecyl sulfatepolyacrylamide gel (10%) electrophoresis (SDS-PAGE). Specific antibodies against BDNF (Mouse monoclonal Anti-BDNF antibody in dilution 1:1000). PSD95 (Rabbit polyclonal Anti-PSD95 antibody, in dilution 1:350), GFAP (Goat polyclonal anti-GFAP antibody, in dilution 1:1000), and GLUR (Rabbit polyclonal Anti-Glutamate receptor 1 antibody (AMPA subtype) antibody, in dilution 1:350) were used for primary immunodetection. Peroxidase-labelled Anti-mouse (in dilution 1:1000), Anti-rabbit (in dilution 1:1000) and Anti-goat (in dilution 1:5000) immunoglobulins were used as the secondary antibodies. Bound antibodies were detected using the enhanced chemiluminescence detection method using Amersham Imager 600 (GE Healthcare), Chicago, IL, USA). Densitometric quantification of protein levels was performed using ImageJ software compared to loading control β-actin for the particulate fraction (dilution 1:1000) and glyceraldehyde-3-phosphate dehydrogenase (GAPDH) for the soluble fraction (in dilution 1:1000) and the corresponding anti-mouse and anti-rabbit secondary antibody.

***Assessment of the hippocampal synaptophysin levels***

Animals were decapitated (between 8:00 am and noon). The brains were extracted (not perfused) and post-fixed with 4 % paraformaldehyde for 24 hrs. cryoprotected in 30 % sucrose/phosphate buffered saline solution for up to 1 week, rapid frozen with liquid nitrogen and kept at -80 °C. Before the immunohistochemical assay, brain tissue was cut into 40 μm sections on a cryostat (Leica). The tissue sections were stored in an antifreeze solution at -15 °C. CA3, CA4, and DG of the dorsal hippocampus were assessed with the endogenous marker synaptophysin (Monoclonal Anti-Synaptophysin, Sigma) as previously described (Gemmel et al., 2018c). Tris-buffered saline (TBS) was used to rinse the sections between individual steps. Free-floating tissue was treated with 0.6 % H2O2 for 30 min at room temperature, then incubated with 5% normal goat serum (NGS) (Lampire Biological Laboratories) in TBS at room temperature for 30 min., followed by overnight incubation with mouse anti-synaptophysin (1:200, Sigma Aldrich) at 4 ºC. The next day, sections were incubated for 2 hrs. in biotinylated rabbit anti-goat secondary antibody (1:500; Vector Laboratories) at room temperature. Brain sections were further processed using the Avidin-Biotin Complex (ABC Elite kit; 1:1000; Vector Laboratories) and the DAB kit (Vector Laboratories). Sections were mounted on Starfrost Advanced Adhesive for IHC (Bamed) dried, dehydrated and cover slipped with Permount (Fisher Scientific).

***Quantification of the optical densities of synaptophysin***

Sections of the dorsal hippocampus were analyzed for optical densities of synaptophysin. Immunoreactivity for all sections was examined under a 40x objective using Leica DM4000M microscope. Photomicrographs were taken for three areas within each hippocampal analyzed region, i.e., CA3, CA4 and DG. ImageJ64 software (Wayne Rasband, NIH, Bethesda, MD, USA) was used for the evaluation of optical densities for all immunoreactive cells. The relative optical density was defined as the difference in optical density (grey level) after calibration between the area of interest and the background, which was an equivalent area adjacent to the area of interest with minimal staining.

***Electrophysiology in vivo***

*In vivo* electrophysiological experiments were carried out on male and female offspring of antidepressant or vehicle treated CUS or non-CUS dams who had reached the age of 48-56 days, as described previously^5^. The animals were anaesthetized with chloral hydrate (400 mg/kg, i.p.) and placed in the stereotaxic frame (David Kopf Instruments, Tujunga, CA). A heating pad was used to maintain the body temperature at between 36 and 37°C (Gaymor Instruments, Orchard Park, NY, USA). The scalp was opened, and a 3 mm hole was drilled in the skull for insertion of electrodes. The electrodes were inserted into the CA1/3 of the hippocampus (3.9-4.2 mm posterior to bregma, 2.2-2.8 mm lateral to the midline, and 1.9-3.5 mm ventral to brain surface), DRN (7.8-8.3 mm posterior to bregma and 4.5-7.0 mm ventral to the brain surface), and VTA (4.5-5.5 mm posterior to bregma, 0.6-0.8 mm lateral to the midline, and 7.0-8.5 mm ventral to the brain surface)^6^ by a hydraulic micro-positioner (David Kopf Instruments, Tujunga, CA). The action potentials generated by 5-HT neurons were recorded using the AD Instruments Extracellular Recording System (Dunedin, New Zealand).

Glutamate neurons of the hippocampus were identified based on the following criteria: large amplitude (0.5–1.2 mV), long-duration (0.8–1.2 ms) simple action potentials alternating with complex spike discharges^7, 8^. The 5-HT neurons were identified by bi- or tri-phasic action potentials with a rising phase of long duration (0.8–1.2 ms) and regular firing rate of 0.5–5.0 Hz ^9, 10^. The 5-HT neurons were identified by bi- or tri-phasic action potentials with a rising phase of long duration (0.8–1.2 ms) and regular firing rate of 0.5–5.0 Hz^9, 10^. Noradrenaline LC neurons were recognized by action potentials with a long-duration rising phase (0.8-1.2 ms), regular firing rate of 0.5–5.0 Hz, and a characteristic burst discharge in response to nociceptive pinch of the contralateral hind paw^9^. Dopamine neurons were recognized by tri-phasic action potentials lasting between 3 and 5 ms with a rising phase lasting over 1.1 ms, inflection or “notch” during the rising phase, marked negative deflection, irregular firing-rate of 0.5-10 Hz, mixed single-spike and burst firing with characteristic decrease of the action potentials amplitude within the bursts^11^.

***Expression and activity of the RyR2***

The brain endoplasmic reticulum (ER) microsomes enriched in RyR2 channels were isolated from rat subcortical structures located beneath the cerebral cortex, following isolation protocol described by Bilmen and Michelangeli ^14^. For each group, ER microsomes were isolated from brain tissue of 2–3 animals, with two isolations performed per group. Planar lipid membranes (BLMs) of a 3:1 mixture of 1,2-dioleoyl-sn-glycero-3-phosphoethanolamine (DOPE) and 1,2-dioleoyl-sn-glycero-3-phosphocholine (DOPC) were formed across a 50-80 µm circular aperture in the wall of a polystyrene cup that separates two compartments, cytosolic and luminal. Phospholipids DOPE and DOPC were obtained from Avanti Research (Alabaster, AL). The cytosolic compartment was filled with 1 mL of 150 mM KCl, 10 mM Tris and ~20 mM HEPES (pH = 7.35). The free cytosolic Ca^2+^ concentration of 90 nM was obtained by including 1 mM ethylene glycol-bis(β-aminoethylether)–N,N,N′,N′-tetraacetic acid (EGTA) and 0.544 mM CaCl_2_. The free Ca^2+^([Ca^2+^]_C_) concentrations were determined by WinMaxc32 version 2.50 (<http://www.stanford.edu/~cpatton/maxc.html>). The luminal compartment was filled with 1 mL of 8 mM Ca(OH)_2_, 150 mM KCl, 10 mM Tris and ~22 mM HEPES (pH = 7.35). The current in the physiological relevant direction (from lumen to cytosol) was carried by Ca^2+^ ions and activity of RyR2 channels incorporated into the BLMs were recorded under voltage-clamp conditions. The luminal compartment was connected to the head-stage input of an Axopatch 200B amplifier (Molecular Devices, Sunnyvale, CA) and the cytosolic compartment was held at virtual ground. In all experiment, the holding potential was 0 mV. Electrical signals were filtered through an Axopatch 200B low-pass Bessel filter at 1 kHz, digitized at 4 kHz with an A/D-D/A converter (Digidata 1550A, Molecular Devices, Sunnyvale, CA). Data acquisition and analysis were performed with a commercially available software pCLAMP 10.5 (Molecular Devices, Sunnyvale, CA). The open probability (P_O_), as a measure of channel activity, was calculated from continuous records of >3 min in duration using the 50%-amplitude threshold method. P_O_ was calculated for individual ion channels under resting conditions ([Ca^2+^]_C_ =90 nM). ER microsomal preparations (45-55 µg), the same as used for RyR2 single-channel recordings, were subjected to 3–8% SDS-PAGE and wet transferred onto PVDF membrane. Immunoblots were developed with the following primary antibodies: anti-RyR (Invitrogen, 1:5000), anti-phospho-RyR2-pSer2808 (Badrilla Ltd., 1:2500) and anti-β-actin (Invitrogen, 1:10000). β-actin served as an internal control to correct for sample loading error. GelAnalyzer 23.1.1 software (available at www.gelanalyzer.com).

**Figure S1:** Western blot analysis of RyR2 channels isolated from subcortical structures of the rat brain from male and female offspring of dams which were unstressed-control (C), unstressed-control and treated with mirtazapine (C+Mir), stressed (CUS) and stressed and treated with mirtazapine (CUS+Mir). (**A**) Representative immunoblots and quantification of total RyR2 level for male and female offspring (**B**) Representative immunoblots and evaluation of RyR2 phosphorylation at Serine 2808 for male and female offspring for the total RyR2 level, signal intensities were normalized to β-actin and then to male control (means ± SEM obtained from two ER microsomal preparations from brain tissue of 2–3 animals analyzed in triplicate). For evaluation of RyR2 phosphorylation, signal intensities were normalized to β-actin and then to corresponding male or female control (mean ± SEM obtained from one ER microsomal preparations from brain tissue of 2–3 animals analyzed in triplicate). (**C**) Representative current traces of RyR2 channels from male and female offspring and statistical analysis of the resting RyR2 P_O_ (measured at [Ca^2+^]_C_=90 nM). Data are normalized to male control and are displayed as mean ± SEM (n= 5−7).

**References**

1. Bogi E, Belovicova K, Moravcikova L, Csatlosova K, Dremencov E, Lacinova L *et al.* Pre-gestational stress impacts excitability of hippocampal cells in vitro and is associated with neurobehavioral alterations during adulthood. *Behav Brain Res* 2019; 375: 112131.

2. Viñas-Noguera M, Csatlósová K, Šimončičová E, Bögi E, Ujházy E, Dubovický M *et al.* Sex- and age- dependent effect of pre-gestational chronic stress and mirtazapine treatment on neurobehavioral development of Wistar rat offspring. *PLoS One* 2022; 17(2): e0255546.

3. Maková M, Kašparová S, Tvrdík T, Noguera M, Belovičová K, Csatlosová K *et al.* Mirtazapine modulates Glutamate and GABA levels in the animal model of maternal depression. MRI and (1)H MRS study in female rats. *Behav Brain Res* 2023; 442: 114296.

4. Grinchii D, Janáková Csatlósová K, Viñas-Noguera M, Dekhtiarenko R, Paliokha R, Lacinová Ľ *et al.* Effects of pre-gestational exposure to the stressors and perinatal bupropion administration on the firing activity of serotonergic neurons and anxiety-like behavior in rats. *Behav Brain Res* 2024; 459: 114796.

5. Csatlosova K, Bogi E, Durisova B, Grinchii D, Paliokha R, Moravcikova L *et al.* Maternal immune activation in rats attenuates the excitability of monoamine-secreting neurons in adult offspring in a sex-specific way. *Eur Neuropsychopharmacol* 2021; 43: 82-91.

6. Paxinos G, Watson C. *Paxino's and Watson's The rat brain in stereotaxic coordinates*. Seventh edition. edn. Elsevier/AP, Academic Press is an imprint of Elsevier: Amsterdam ; Boston, 2014, 1 volume (unpaged)pp.

7. El Mansari M, Ebrahimzadeh M, Hamati R, Iro CM, Farkas B, Kiss B *et al.* Long-term administration of cariprazine increases locus coeruleus noradrenergic neurons activity and serotonin(1A) receptor neurotransmission in the hippocampus. *J Psychopharmacol* 2020; 34(10): 1143-1154.

8. Kandel ER, Spencer WA. Electrophysiology of hippocampal neurons. II. After-potentials and repetitive firing. *J Neurophysiol* 1961; 24: 243-259.

9. Vandermaelen CP, Aghajanian GK. Electrophysiological and pharmacological characterization of serotonergic dorsal raphe neurons recorded extracellularly and intracellularly in rat brain slices. *Brain Res* 1983; 289(1-2): 109-119.

10. Koprdova R, Csatlosova K, Durisova B, Bogi E, Majekova M, Dremencov E *et al.* Electrophysiology and Behavioral Assessment of the New Molecule SMe1EC2M3 as a Representative of the Future Class of Triple Reuptake Inhibitors. 2019; 24(23): 4218.

11. Grace AA, Bunney BS. Intracellular and extracellular electrophysiology of nigral dopaminergic neurons--1. Identification and characterization. *Neuroscience* 1983; 10(2): 301-315.

12. Dremencov E, Grinchii D, Hrivikova K, Lapshin M, Komelkova M, Graban J *et al.* Exposure to chronic stressor upsurges the excitability of serotoninergic neurons and diminishes concentrations of circulating corticosteroids in rats two weeks thereafter. *Pharmacol Rep* 2022.

13. Grinchii D, Hoener MC, Khoury T, Dekhtiarenko R, Nejati Bervanlou R, Jezova D *et al.* Effects of acute and chronic administration of trace amine-associated receptor 1 (TAAR1) ligands on in vivo excitability of central monoamine-secreting neurons in rats. *Mol Psychiatry* 2022; 27(12): 4861-4868.

14. Bilmen JG, Michelangeli F. Inhibition of the type 1 inositol 1,4,5-trisphosphate receptor by 2-aminoethoxydiphenylborate. *Cellular Signalling* 2002; 14: 955-960.
